# Preoperative prediction of lymph node metastasis in patients with ovarian cancer using contrast-enhanced computed tomography-based intratumoral and peritumoral radiomics features

**DOI:** 10.3389/fonc.2025.1543873

**Published:** 2025-05-14

**Authors:** Jing Zhang, Qiyuan Li, Haoyu Liang, Yao Wang, Li Sun, Qingyuan Zhang, Chuanping Gao

**Affiliations:** ^1^ Department of Radiology, Affiliated Hospital of Qingdao University, Qingdao, China; ^2^ Huashan Hospital, Fudan University, Shanghai, China

**Keywords:** ovarian cancer, lymph node metastasis, radiomics, tomography - methods, x-ray computed

## Abstract

**Purpose:**

To develop and validate computed tomography (CT)-based intratumoral and peritumoral radiomics signatures for preoperative prediction of lymph node metastasis (LNM) in patients with ovarian cancer (OC).

**Methods:**

Patients with pathological diagnosis of OC were retrospectively included. Intratumoral and peritumoral radiomics features were extracted from contrast-enhanced CT images. Intratumoral and peritumoral radiomics features were extracted from contrast-enhanced CT images. Intratumoral, peritumoral, and combined radiomics signatures were constructed, and their radiomics scores were calculated. Univariate and multivariate logistic regression analyses were performed to identify predictors of clinical outcomes. A radiomics nomogram was developed by incorporating the combined radiomics signature with clinical risk factors. The prediction efficiency of the various models was evaluated using the accuracy value, the area under the receiver-operating characteristic curve (AUC) and decision curve analysis (DCA).

**Results:**

Two hundred and seventy-three patients with OC were enrolled and randomly divided into a training cohort (n=190) and a test cohort (n=83) in a 7:3 ratio. The intratumoral, peritumoral, and combined radiomics signatures were constructed using 18, 11, and 17 radiomics features, respectively. The combined radiomics signature showed the best prediction ability, with accuracy of 0.783 and an AUC of 0.860 (95% confidence interval 0.779–0.941). The DCA results showed that the combined radiomics signature had better clinical application than the clinical model and the radiomics nomogram.

**Conclusions:**

A CT-based combined radiomics signature incorporating intratumoral and peritumoral radiomics features can predict LNM in patients with OC before surgery.

## Introduction

Ovarian cancer (OC) has the highest mortality of all the gynecological malignancies, with a 5-year survival rate below 45% (1). Approximately 22,400 new cases are reported each year in the US, less than 40% of which are amenable to curative treatment (2). Surgical resection is considered the most important treatment for OC but the role of systemic lymphadenectomy remains controversial. Lymph node (LN) status may affect survival in these patients and is included in the International Federation of Gynecology and Obstetrics (FIGO) staging system (3). OC with lymph node metastasis (LNM) is typically classified as stage III or higher (4). Accurate identification of LNM enables clinicians to plan appropriate treatment. However, there is still no reliable methods for predicting LNM preoperatively in patients with OC.

Imaging examinations are widely used for detection and staging of OC. Computed tomography (CT) is recommended by the European Society of Urogenital Radiology as the first-line imaging modality for preoperative staging of OC and follow-up and has the advantages of a fast scanning time and good reproducibility (5). However, its accuracy for preoperative imaging assessment of LNM has been unsatisfactory, with a sensitivity of only 48%–80% (6). Radiomics, a process by which medical images are converted into mineable high-dimensional data in a high-throughput manner, has recently been introduced in the medical imaging field for the purposes of preoperative diagnosis (7, 8). The resulting data can provide information about the predicted severity of a given disease, the associated risks, and the likely response to therapy and can be used to support clinical decision-making. Radiomics is capable of characterizing the heterogeneity of an entire tumor and its microenvironment and has the advantages of being non-invasive and not constrained by time or space (9, 10). Introduction of radiomics has been found to improve the prediction of preoperative LNM, disease stage, post-treatment response, and survival in patients with colorectal, thyroid, cervical, and cholangiocarcinoma tumors (11–14). To the best of our knowledge, most of the radiomics studies in OC have focused on differential diagnosis and preoperative staging of the disease (15, 16), but few have focused on preoperative prediction of LNM and have been based on intratumoral radiomics features (17). However, peritumoral radiomics features have also demonstrated predictive value in several types of cancers (18, 19). Therefore, in this study, we sought to evaluate the efficiency of intratumoral and peritumoral radiomics in preoperative prediction of LNM in patients with OC.

## Materials and methods

### Patients

The study was approved by the Institutional Review Board of the Affiliated Hospital of Qingdao University. The requirement for informed consent was waived in view of the anonymity of the data analyzed and non-interventional nature of the research.

We searched the institutional pathology database from October 2016 to March 2023 and identified 1,117 patients with a diagnosis of OC based on surgically resected specimens who underwent appropriate surgery with pelvic and/or para-aortic LN dissection. The inclusion criteria were pathologically confirmed OC and a contrast-enhanced CT examination within the 2 weeks before surgery. Patients with other malignancies, those who had received preoperative chemoradiotherapy, and those with images of insufficient quality were excluded.

The following clinical and pathological data were collected: age, tumor size, pathological type, FIGO stage, carbohydrate antigen 125 (CA125) and human epididymal protein 4 (HE4) levels, presence or absence of ascites, and CT-reported LN status.

### CT image acquisition and radiological evaluation

Contrast-enhanced CT was performed in all patients. The helical CT systems used were the Somatom Sensation 64 and Somatom Definition (Siemens Healthcare, Erlangen, Germany) and the Discovery 750 and Brightspeed 16 (GE Healthcare, Little Chalfont, UK). The scan area was from the pubic symphysis to the diaphragm, and the lesions were observed in the soft-tissue window. The CT scan parameters were as follows: tube current, 240–320 mAs; tube voltage, 120 kVp; beam pitch, 1.375; matrix, 512×512; and section thickness, 5 mm. An intravenous non-ionic contrast agent (Ultravist 370 [iopromide]; Bayer, Berlin, Germany) were used in all patients at a rate of 3 ml/s and a dose of 1.5 ml/kg patient body weight. The respective arterial, venous, and delayed phase times were 30 s, 60–70 s, and 120 s after injection of contrast.

Two radiologists experienced in gynecological imaging reviewed all CT images and assessed the following characteristics: tumor size (maximum diameter on transverse images); CT-reported ascites and LN status; and LN status. A maximum short diameter of >10 mm in the portal vein phase was defined as positive for LNM (20). The two radiologists were blinded to the pathology data. Any disagreements were resolved by consultation.

### Image segmentation

**Figure 1:**
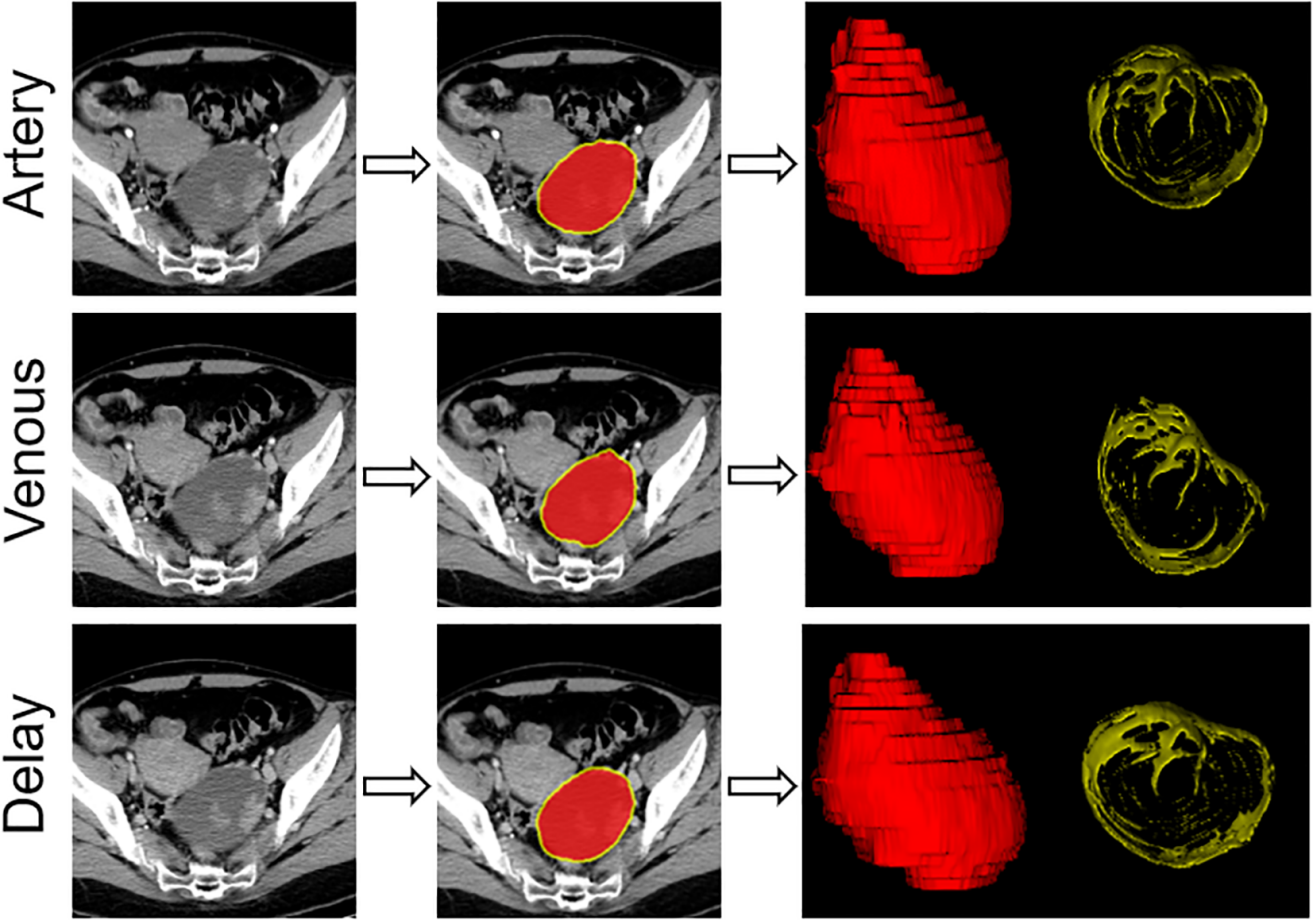
Flowchart showing the process used for segmentation of contrast-enhanced computed tomography images with an example of primary tumor segmentation for a patient with ovarian cancer.

A schematic of image segmentation is shown in **Figure 1**. The two radiologists used open-source imaging software (ITK-SNAP, version 3.8.0; www.itksnap.org) for three-dimensional segmentation. The tumor regions were segmented along the tumor contour on each enhanced axial CT image slice as the intratumoral region of interest (ROI). The peritumoral ROI was generated using the “ROI Operation” module in RIAS software (21), which automatically extended 3 mm outwards from the tumor and removed the tumor area.

### Extraction of radiomics features

The radiomics features were extracted using a 3D-Slicer (version 5.3.0, https://www.slicer.org). Given that the images were acquired using various scanners, the CT images were resampled, grayscale discretized, and normalized before feature extraction. The radiomics features were extracted from both the original intratumoral and peritumoral ROIs of all phase images. In total, 6780 (3390 + 3390) radiomics features were extracted.

### Intraobserver and interobserver reproducibility

Intraclass correlation coefficients (ICCs) were calculated to assess intraobserver and interobserver reproducibility before selection of features. Initially, 30 CT images were randomly selected and segmented by reader 1 and reader 2 to assess interobserver reproducibility. Reader 1 repeated the segmentation 2 weeks later for assessment of intraobserver reproducibility. Features with an ICC >0.75 were considered to have good reproducibility and included. The remaining segmentations were performed independently by reader 1.

### Development and evaluation of intratumoral, peritumoral, and combined radiomics signatures

Selection of radiomics features proceeded in two steps, with the same process used to select both intratumoral and peritumoral radiomics features. First, the minimum redundancy maximum correlation algorithm was used to select the top 30 features. Second, the least absolute shrinkage and selection operator algorithm was used to determine the penalty parameter through 10-fold cross-validation, after which the optimal features were selected. The radiomics score for each patient was calculated as a linear combination of selected features, which were weighted by their respective coefficients. The area under the curve (AUC) of the receiver-operating characteristic (ROC) curve was calculated to evaluate the performance of the three radiomics signatures for prediction of LNM in the training cohort, which was confirmed in the test cohort.

### Development of a clinical model and radiomics nomogram

The relationship between LN status and clinical parameters was evaluated by univariate logistic regression analysis. Clinical risk factors with a p-value <0.05 were then subjected to multivariate logistic regression analysis to develop the clinical model. A radiomics nomogram was developed by incorporating the best-performing radiomics signature and clinical risk factors.

### Assessment of performance of the radiomics signature, clinical model, and radiomics nomogram

The ability of the radiomics signature, clinical model, and radiomics nomogram to predict LNM was evaluated by the AUC and accuracy values. Calibration curves were plotted to assess the consistency between the predictions of the nomogram and the observed outcomes. Decision curve analysis (DCA) was used to evaluate the clinical value of the various models.

### Statistical analysis

All statistical analyses were performed using R statistical software (version 4.2.2, http://www.r-project.org). A two-sided p-value of <0.05 was considered statistically significant. Continuous variables were compared between groups using the Student’s *t*-test and Mann–Whitney *U* test, and class-based variables were compared using the chi-squared test. The DeLong test was used to assess differences in the AUC among the models. The univariate and multivariate logistic analyses were performed using SPSS version 26.0 software (IBM Corp., Armonk, NY, USA).

## Results

### Clinical characteristics

**Figure 2:**
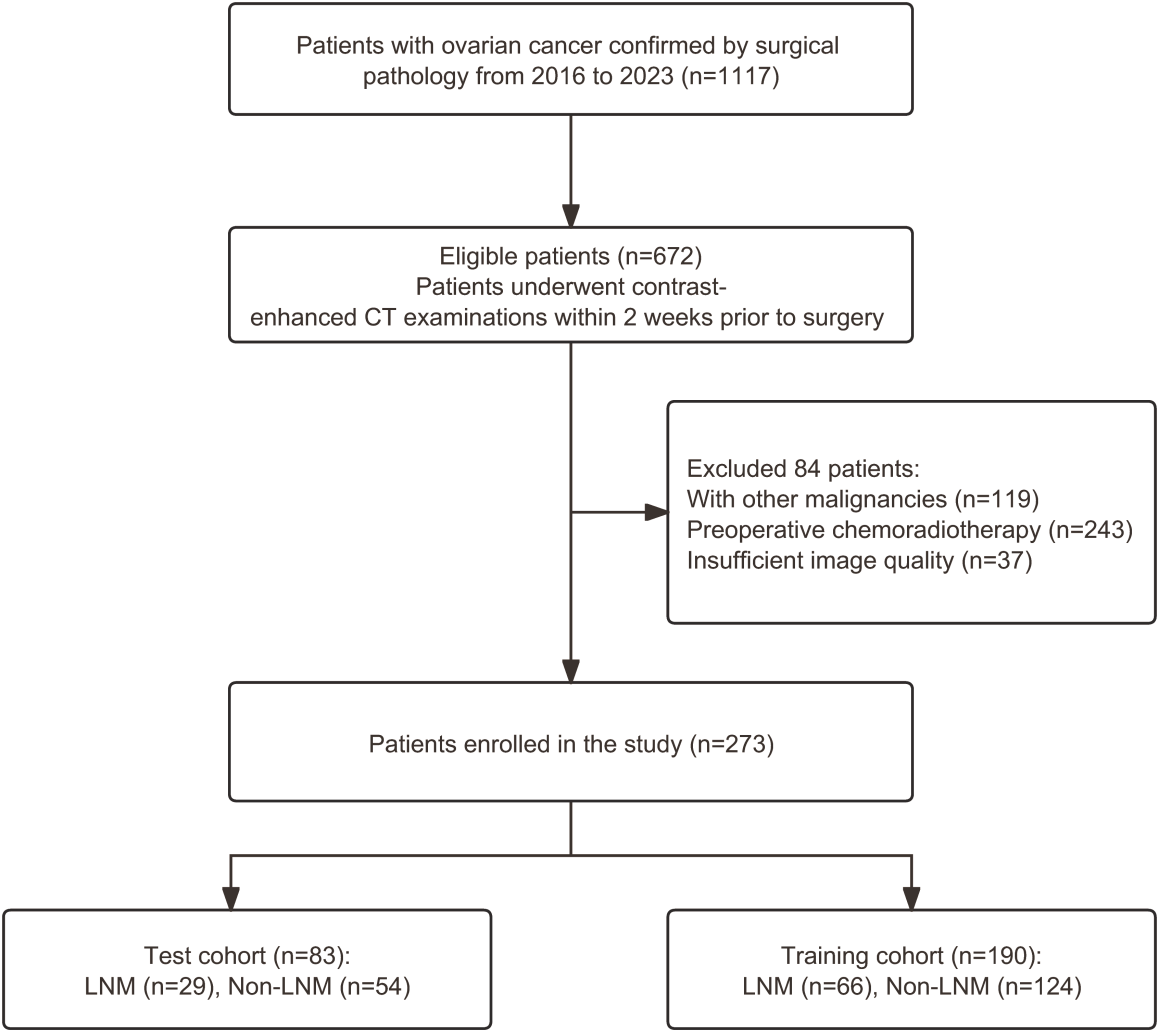
Flow diagram showing the patient selection process. LNM, lymph node metastasis.

A total of 273 patients were enrolled and randomly divided into a training cohort (n=190) and a test cohort (n=83) in a 7:3 ratio. A flowchart showing the patient recruitment process is provided in **Figure 2**.

**Table 1** shows the clinical and pathological characteristics of the 273 patients with OC. The median patient age was 53 years (range 19–80). Metastasis was confirmed intraoperatively in 95 patients (34.8%).

**Table 1:** Patient’s Clinical and pathological information between metastasis and non-metastasis group in the training and test cohort.

Variables	Training cohort (n=190)			Test cohort (n=83)		
	LNM (n=66)	non-LNM (n=124)	P	LNM (n=29)	Non-LNM (n=54)	P
Age (years), mean ± SD	54.48±10.15	53.83±11.48	0.654	51.13±8.17	52.81±9.99	0.441
Tumor size, median (IQR)	92.00 (60.00,125.50)	103.50 (74.00,137.25)	0.043	83.00 (67.50,115.00)	92.50 (74.25,128.50)	0.202
Pathological type						
High-grade serous adenocarcinoma	54	60	0.001	25	28	0.047
Low-grade serous adenocarcinoma	3	2		2	4	
Clear cell carcinoma	3	22		2	13	
Endometrioid cancer	4	13		0	4	
Mucinous carcinoma	3	13		0	2	
Other	2	11		0	2	
FIGO stage						
I	1	61	<0.001	4	27	<0.001
II	3	26		0	12	
III	53	31		17	12	
IV	9	6		8	3	
CA-125, median (IQR)	560.40 (162.74,1377.25)	120.95 (44.70,510.60)	<0.001	378.00 (131.75,1301.00)	170.95 (38.02,504.90)	0.022
HE4, median (IQR)	268.20 (147.40,461.45)	116.20 (64.11,236.50)	<0.001	222.10 (153.10,405.00)	105.50 (58.25,181.28)	<0.001
CT-reported LN status						
LNM	28	11	<0.001	15	8	0.001
non-LNM	38	113		14	46	
CT-reported ascites						
Present	47	77	0.263	24	29	0.016
Absent	19	47		5	25	

SD, Standard Deviation; IQR, Interquartile Range; LNM, Lymph Node Metastasis; FIGO, International Federation of Gynecology and Obstetrics.

### Selection of features and construction of intratumoral, peritumoral, and combined radiomics signatures

In total, 1,628 intratumoral and 1,858 peritumoral radiomics features showed high stability (ICC >0.75). Finally, 18, 11, and 17 radiomics features, respectively, were selected using the mRMR and LASSO algorithms to construct intratumoral, peritumoral, and combined radiomics signatures (RS-region, RS-peri, RS-combined). The formula used to calculate the radiomics score is shown in the **Supplementary Methods**.

**Figure 3:**
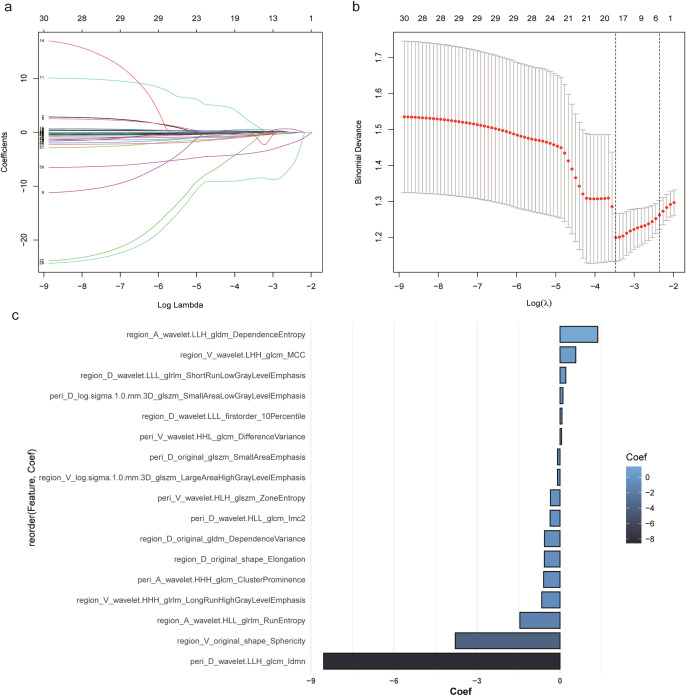
Selection of features for development of the combined radiomics signature. **(a)** Coefficients drawn vs. ln (λ). **(b)** Selection process for tuning parameter (λ). **(c)** The 17 radiomics features selected.

**Table 2** shows the accuracy, AUC, and confidence interval (CI) for each of the models. Of the three radiomics signatures, the RS-combined showed the best predictive performance and was used to construct a radiomics nomogram with an AUC of 0.860 and accuracy of 0.783 in the test cohort. **Figure 3** shows the process used to select features for development of the RS-combined.

**Table 2:** Performance of clinical model, radiomics signatures and radiomics nomogram.

Model	Training cohort				Test Cohort			
	AUC (95%CI)	ACC	SEN	SPE	C-index (95%CI)	ACC	SEN	SPE
Clinical model	0.850 (0.899-0.901)	0.742	0.455	0.895	0.600 (0.472-0.728)	0.602	0.345	0.741
Radiomics nomogram	0.925 (0.891-0.960)	0.805	0.697	0.863	0.870 (0.782-0.957)	0.675	0.862	0.574
RS _combine_	0.850 (0.792-0.908)	0.795	0.424	0.992	0.860 (0.779-0.941)	0.783	0.621	0.870
RS _region_	0.778 (0.711-0.846)	0.711	0.439	0.855	0.579 (0.451-0.706)	0.590	0.414	0.685
RS _peri_	0.749 (0.678-0.821)	0.705	0.394	0.871	0.579 (0.451-0.706)	0.590	0.310	0.741

RS, radiomics signature; AUC, area under the curve; CI, confidence interval; ACC, accuracy; SEN, sensitivity; SPE, specificity.

### Development of the clinical model

The results of the univariate and multivariate logistic regression analyses of clinical characteristics are shown in **Table 3**. In univariate analysis, tumor size, pathological type, CA125, HE4, FIGO stage, and CT-reported LN status were significant risk factors for LNM (p<0.05). However, in multivariate analysis, only FIGO stage (odds ratio [OR] 4.041; 95% CI 2.387–8.117; p<0.001) and CT-reported LN status (OR 0.294; 95% CI 0.119–0.726; p=0.008) were independent risk factors and were included in the clinical model. The AUC for the clinical model was 0.850 (95% CI 0.799–0.901) in the training cohort and 0.600 (95% CI 0.472–0.728) in the test cohort (**Table 2**).

**Table 3:** Results of univariate and multivariate logistic regression analysis in ovarian cancer patients.

Variables	Univariate Logical Analysis		Multivariate Logical Analysis	
	OR (95%CI)	P	OR (95%CI)	P
Age	1.005 (0.978-1.033)	0.696		
Tumor size	0.992 (0.984-0.999)	0.031	0.998 (0.987-1.010)	0.198
Pathological type	0.587 (0.457-0.753)	<0.001	0.823 (0.612-1.107)	0.783
FIGO stage	5.855 (3.353-10.226)	<0.001	4.401 (2.387-8.117)	<0.001
CA-125	1.000 (1.000-1.001)	0.024	1.000 (1.000-1.000)	0.389
HE4	1.001 (1.000-1.002)	0.013	1.000 (0.999-1.001)	0.854
CT-reported LN status	0.132 (0.060-0.291)	<0.001	0.294 (0.119-0.726)	0.008
CT-reported ascites	0.662 (0.348-1.262)	0.210		

OR, odds ratio; CI, confidence interval.

### Construction of a radiomics nomogram and performance of the various models

**Figure 4:**
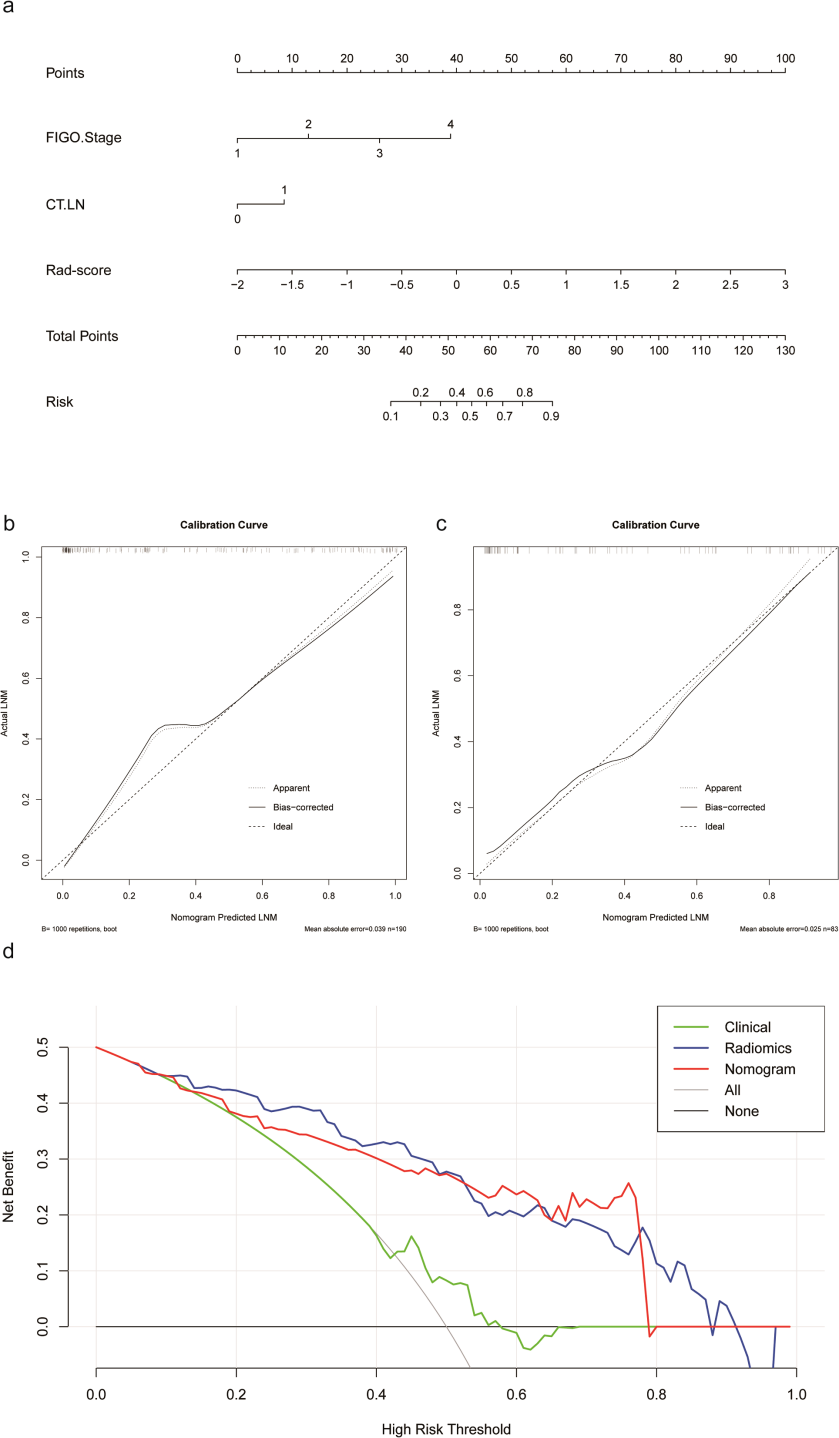
**(a)** Radiomics nomogram. **(b)** Calibration curve of the radiomics nomogram for the training cohort. **(c)** Calibration curve of the radiomics nomogram for the test cohort. **(d)** Decision curve analysis of the various models.

A radiomics nomogram was developed by incorporating the RS-combined and independent clinical factors (**Figure 4a**). The AUC was 0.925 (95% CI 0.891–0.960) in the training cohort and 0.870 (95% CI 0.782–0.957) in the test cohort (**Table 2**). The calibration curve (**Figures 4b, c**) showed that the radiomics nomogram-based predictions were in good agreement with the true LNM status.

As shown in **Table 2**, the AUC of the RS-combined was significantly higher in the test cohort than in the clinical model (p<0.001) but was not significant different from that in the radiomics nomogram (p=0.862). However, the training accuracy of the RS-combined was significantly higher than that of the radiomics nomogram and the clinical model (0.783 vs.0.675 vs. 0.602).

The DCA curves for the RS-combined, clinical model, and radiomics nomogram in the test cohort are shown in **Figure 4d**. The RS-combined showed markedly better performance than the radiomics nomogram in most threshold range and consistently outperformed the clinical model.

## Discussion

In this study, we developed various radiomics signatures, a clinical model, and a radiomics nomogram based on radiomics and clinical features for preoperative prediction of LNM in patients with OC. Our results show that a radiomics signature that integrated intratumoral and peritumoral radiomics features had the best predictive efficiency with the highest AUC values and accuracy.

Systemic lymphadenectomy is essential for accurate staging, prognosis, and assessment of the effects of oncological treatment. Previous studies have found that the average incidence of LNM is 44%–53% in patients with FIGO stage III–IV OC (22), in whom lymphadenectomy can improve 5-year overall survival. However, lymphadenectomy failed to improve 5-year overall survival in patients with FIGO stage I–II, and the incidence of LNM in these patients was reported to be only 14.2% (23), suggesting that overtreatment occurs in at least 80% of cases. In this study, 95 (34.8%) of 273 patients were confirmed to have LNM, which is consistent with the value of 36.8% reported by Xiang et al. (24). Therefore, accurately identifying the risk of LNM preoperatively has clinical significance in the treatment of OC.

Controversy persists regarding the risk factors for LNM in patients with OC. Zhou et al. identified three independent risk factors for LNM in patients with OC, namely, histological type, grade, and CA125 level at diagnosis. They constructed a predictive model using these factors and reported an AUC of 0.740 (4), which suggests that clinical parameters for preoperative assessment of LN status are still not accurate enough. In recent years, radiomics has been confirmed to be useful for preoperative prediction of LN status in certain tumors. Liu et al. developed a CT-based radiomics nomogram for prediction of LNM in gallbladder cancer and found that the radiomics score was an independent predictor of LNM (OR 7.415; 95% CI 3.384–16.246; p<0.001) and that addition of radiomics analysis significantly improved the accuracy of prediction (25). CT is the standard imaging modality for preoperative evaluation and postoperative surveillance of patients with OC, and LNM is mainly evaluated by measuring LN size (26). Ai et al. constructed a model for prediction of LNM in patients with OC that combined nine non-contrast-enhanced CT-based radiomics features and two clinical factors (age and CA125). The model had good prediction value with an AUC of 0.86 (95% CI 0.72–0.99), sensitivity of 0.81, and specificity of 0.8 (27).

Peritumoral heterogeneity and the microenvironment are closely related to tumor aggressiveness. The peritumoral area reflects infiltration of peritumoral immune cells (28, 29), and changes in the stroma surrounding the tumor determine the ability of the tumor to grow and spread, evade the body’s immune protection system, and resist therapeutic intervention (30). In recent years, many studies have incorporated peritumoral radiomics features into intratumoral radiomics or clinical models. These models have often been used for survival analysis and differential diagnosis in patients with cancer and for preoperative prediction (18, 19, 31). Wang et al. found that both peritumoral and intratumoral radiomics features had good predictive performance for LNM in patients with lung adenocarcinoma (AUC 0.825 vs. 0.829). Moreover, when they integrated intratumoral and peritumoral radiomics features with clinical parameters, the predictive ability was improved further (AUC 0.863, 95% CI 0.800–0.938) (19). Yang et al. assessed the relationship between preoperative imaging data and LNM in 193 patients with gastric cancer and found that a model that integrated intratumoral and peritumoral radiomics features had better predictive ability than a model based on intratumoral radiomics features (AUC 0.779 vs. 0.717) (32). To the best of our knowledge, no studies have integrated intratumoral and peritumoral radiomics features for prediction of LNM in patients with OC.

We have constructed intratumoral, peritumoral, and combined radiomics signatures based on contrast-enhanced CT and demonstrated that the combined radiomics signature shows better predictive performance than the intratumoral radiomics signature and peritumoral radiomics signature (AUC 0.860 vs.0.579 vs. 0.579). Peritumoral radiomics might contain unique and valuable information that has additional predictive value for LNM in patients with OC.

This study has several limitations. First, it had a single-center retrospective design with the inherent risk of selection bias. Multicenter studies with larger sample sizes are needed to improve the robustness of the model. Second, segmentation of tumors was performed manually, which can be time-consuming, laborious, and error-prone. Further research is necessary to improve precision of segmentation and to automate the segmentation process. Third, the radiomics features were extracted from the primary tumor instead of LNs, which may affect the accuracy of the model. Image acquisition, feature extraction, and data processing should be standardized before implementation of radiomics analysis in clinical practice.

## Conclusion

A combined radiomics signature that incorporates intratumoral and peritumoral radiomics features could serve as an effective tool for preoperative prediction of LNM in patients with OC, thereby guiding clinical decision-making and facilitating individualized management of these patients.

## Data Availability

The datasets presented in this study can be found in online repositories. The names of the repository/repositories and accession number(s) can be found in the article/**Supplementary Material**.
