# Supplementary material for: Preoperative prediction of lymph node metastasis in patients with ovarian cancer using contrast-enhanced computed tomography-based intratumoral and peritumoral radiomics features
Source: Front Oncol. 2025 May 14;15:1543873. doi: 10.3389/fonc.2025.1543873 (PMC12116341; doi:10.3389/fonc.2025.1543873)
Supplement: Supplementary file 1 [file DataSheet1.docx]

**Supplementary Materials**

The formula of the intratumoral radiomics score is as follows.

Rad-score= 35.223-0.095×region_V_wavelet.LHL_ngtdm_Busyness+ 0.375×region_V_log.sigma.0.5.mm.3D_glszm_GrayLevelNonUniformityNormalized+0.438×region_D_wavelet.LLL_glrlm_ShortRunLowGrayLevelEmphasis-

0.157×region_V_log.sigma.1.5.mm.3D_glrlm_RunVariance+

3.822×region_A_wavelet.LLH_gldm_DependenceEntropy+

0.005×region_A_log.sigma.1.5.mm.3D_glszm_LowGrayLevelZoneEmphasis-0.022×region_V_wavelet.HHL_glszm_SmallAreaEmphasis+

0.184×region_D_wavelet.LLL_firstorder_10Percentile-0.798×region_D_original_gldm_DependenceVariance-

0.001×region_D_original_glszm_LargeAreaLowGrayLevelEmphasis-0.598×region_D_original_shape_Elongation-0.142×region_V_log.sigma.1.0.mm.3D_glszm_LargeAreaHighGrayLevelEmphasis-4.791×region_A_wavelet.HLL_glrlm_RunEntropy-1.115×region_V_wavelet.HHH_glrlm_LongRunHighGrayLevelEmphasis+

0.482×region_V_wavelet.LHH_glcm_MCC-29.343×region_D_wavelet.LLL_glcm_Idmn-4.187×region_V_original_shape_Sphericity

The formula of the peritumoral Radiomics score is as follows.

Rad-score=18.583-9.099×peri_A_wavelet.HHH_glcm_ClusterTendency+

0.001×peri_V_wavelet.HLL_glszm_LowGrayLevelZoneEmphasis-

0.002×peri_D_original_glszm_ZoneVariance-

0.283×peri_A_wavelet.HLL_firstorder_90Percentile-

0.354×peri_V_wavelet.HLH_glszm_ZoneEntropy-0.780×peri_D_original_glszm_SmallAreaEmphasis+

0.001×peri_D_original_firstorder_Median-0.112×peri_V_wavelet.LHL_glszm_LargeAreaHighGrayLevelEmphasis+

0.587×peri_D_wavelet.LHH_glcm_MCC-

1.547×peri_D_original_shape_Elongation-

7.80×peri_D_wavelet.LLH_glcm_Idmn

The formula of the combined Radiomics score is as follows.

Rad-score=13.977-0.089×peri_D_original_glszm_SmallAreaEmphasis+

0.108×peri_D_log.sigma.1.0.mm.3D_glszm_SmallAreaLowGrayLevelEmphasis-

0.560×region_D_original_gldm_DependenceVariance+

1.365×region_A_wavelet.LLH_gldm_DependenceEntropy+

0.074×region_D_wavelet.LLL_firstorder_10Percentile+

0.210×region_D_wavelet.LLL_glrlm_ShortRunLowGrayLevelEmphasis-

0.590×peri_A_wavelet.HHH_glcm_ClusterProminence+

0.059×peri_V_wavelet.HHL_glcm_DifferenceVariance-

0.341×peri_V_wavelet.HLH_glszm_ZoneEntropy-0.356×peri_D_wavelet.HLL_glcm_Imc2-0.565×region_D_original_shape_Elongation-

0.092×region_V_log.sigma.1.0.mm.3D_glszm_LargeAreaHighGrayLevelEmphasis-0.661×region_V_wavelet.HHH_glrlm_LongRunHighGrayLevelEmphasis-1.450×region_A_wavelet.HLL_glrlm_RunEntropy+

8.543×peri_D_wavelet.LLH_glcm_Idmn-

3.783×region_V_original_shape_Sphericity
